# **δ**-Tocotrienol Oxazine Derivative Antagonizes Mammary Tumor Cell Compensatory Response to CoCl_**2**_-Induced Hypoxia

**DOI:** 10.1155/2014/285752

**Published:** 2014-07-22

**Authors:** Suryatheja Ananthula, Parash Parajuli, Fathy A. Behery, Alaadin Y. Alayoubi, Sami Nazzal, Khalid El Sayed, Paul W. Sylvester

**Affiliations:** School of Pharmacy, University of Louisiana at Monroe, 700 University Avenue, Monroe, LA 71209-0470, USA

## Abstract

In response to low oxygen supply, cancer cells elevate production of HIF-1*α*, a hypoxia-inducible transcription factor that subsequently acts to stimulate blood vessel formation and promote survival. Studies were conducted to determine the role of *δ*-tocotrienol and a semisynthetic *δ*-tocotrienol oxazine derivative, compound **44**, on +SA mammary tumor cell hypoxic response. Treatment with 150 *µ*M CoCl_2_ induced a hypoxic response in +SA mammary tumor cells as evidenced by a large increase in HIF-1*α* levels, and combined treatment with compound **44** attenuated this response. CoCl_2_-induced hypoxia was also associated with a large increase in Akt/mTOR signaling, activation of downstream targets p70S6K and eIF-4E1, and a significant increase in VEGF production, and combined treatment with compound **44** blocked this response. Additional *in vivo* studies showed that intralesional treatment with compound **44** in BALB/c mice bearing +SA mammary tumors significantly decreased the levels of HIF-1*α*, and this effect was associated with a corresponding decrease in Akt/mTOR signaling and activation of downstream targets p70S6kinase and eIF-4E1. These findings demonstrate that treatment with the *δ*-tocotrienol oxazine derivative, compound **44**, significantly attenuates +SA mammary tumor cell compensatory responses to hypoxia and suggests that this compound may provide benefit in the treatment of rapidly growing solid breast tumors.

## 1. Introduction

Rapidly growing solid tumors often display insufficient blood flow and oxygen deficiency within the deep inner regions of the tumor [1]. Tumor cells are often able to adapt and survive hypoxic conditions by altering their metabolism and phenotypic characteristics [2]. An initial compensatory response to hypoxia in tumor cells is an increased production and accumulation of hypoxia-inducible factor-1 (HIF-1) [3]. HIF-1 is a transcription factor that is involved in promoting cellular survival during adverse hypoxic conditions by altering cellular metabolism to maintain oxygen homeostasis [4, 5]. HIF-1 is a heterodimer consisting of HIF-1*α* and HIF-1*β* subunits [3]. HIF-1*β* is constitutively active in most cells, whereas HIF-1*α* is inducible and is characteristically overexpressed in cancer cells during hypoxic conditions [3].

Under normal conditions, HIF-1*α* is rapidly degraded by ubiquitination, but during hypoxic states, HIF-1*α* becomes stable [6]. HIF-1*α* contains an oxygen-dependent domain that can be modified based on oxygen levels to regulate ubiquitination and degradation of HIF-1*α* [7–9]. In addition, HIF-1*α* translocates into the nucleus of the cell during hypoxic conditions to activate various genes that play a critical role in cell survival [10, 11]. Overexpression of HIF-1*α* is associated with a corresponding large production in VEGF, a growth factor involved in promoting tumor angiogenesis, invasion, and metastasis [12].

The phosphatidylinositol-3-kinase (PI3K)/Akt/mTOR pathway has also been shown to play a critical role in cellular compensatory responses to hypoxia and HIF-1*α* expression [13, 14]. Specifically, mTOR appears to be an important upstream activator of HIF-1*α* [15]. Additional downstream targets for mTOR include p70S6kinase and eukaryotic initiation factor-4E (eIF-4E), which are involved in modulating tumor cell metabolism, apoptosis, and autophagy [16–18]. 4E-BP1 is an endogenous inhibitor that binds to eIF-4E during states of low phosphorylation and inhibits eIF-4E activity [16]. The Ras/Raf/MEK/ERK or MAPK cascade has also been shown to play a role in modulating expression, activity, and posttranslational modification of HIF-1*α* during hypoxic conditions [19].

Previous studies have established that tocotrienols, a subgroup within the vitamin E family of compounds, are potent anticancer agents and *δ*-tocotrienol displays the most potent activity [20–22]. Recently, semisynthetic oxazine derivatives of *δ*-tocotrienol have been shown to possess greater solubility and antiproliferative activity than their natural parent compound against +SA mammary tumor cells grown in cell culture and +SA mammary tumors grown in syngeneic mice [23, 24]. One of the most potent *δ*-tocotrienol oxazine derivatives identified was 12-((R)-6,8-dimethyl-8-((3E,7E)-4,8,12-trimethyltrideca-3,7,11-trienyl)-9,10-dihydrochromeno [5,6-e] [1, 3]oxazin-2(1H,3H,8H)-yl) dodecan-1-ol or compound** 44** [23, 24]. The anticancer activity of *δ*-tocotrienol and its oxazine derivatives were found to be associated with a reduction in PI3K/Akt/mTOR and/or MAPK activity in several types of tumor cells [23, 24]. Furthermore, oxazine derivatives of tocotrienols were found to display significantly greater anticancer potency* in vivo* versus* in vitro*, which appears to be attributed to their greater bioavailability as compared to natural tocotrienols [23, 24].

Studies have shown that treatment with CoCl_2_ can be used to artificially induce hypoxic conditions in cell culture [25]. CoCl_2_ is a chelating agent that traps iron ions and acts to inhibit cellular uptake of oxygen and increased HIF-1*α* expression in tumor cells [26]. Therefore, it was of interest to examine the effects of subeffective antiproliferativedoses of *δ*-tocotrienol and the *δ*-tocotrienol oxazine derivative, compound** 44**, on mouse +SA mammary tumor cell compensatory response to CoCl_2_-induced hypoxia in both cell culture and in the syngeneic mouse mammy tumor model. Subeffective antiproliferative doses of *δ*-tocotrienol and compound** 44 **were selected for use in the present investigation because high doses of these agents were found to initiate apoptosis and induce cancer cell death [23, 24], which would confound the interpretation of the results regarding these agents effects on tumor cell compensatory response to CoCl_2_-induced hypoxia.

## 2. Materials and Methods

### 2.1. Reagents and Antibodies

All reagents were purchased from Sigma Chemical Company (St. Louis, MO, USA) unless otherwise stated. Isolated *δ*-tocotrienol (>95% purity) was generously provided by First Tech International Ltd. (Hong Kong). Antibodies for Akt (#9272), p-Akt (#9271, Ser473), PI3K (#4225), p-mTOR (#2971, Ser2448), mTOR (#2989), p-ERK 1/2 (#4337, Thr202/Tyr204), MEK (#8727), p-MEK 1/2 (#2338, Ser221/217), and *α*-tubulin (#2125) were purchased from Cell Signaling Technology (Beverly, MA, USA). Antibodies for HIF-1*α* (#Sc-8711), ERK1 (#Sc-93), and ERK2 (#Sc-154) were purchased from Santa Cruz Biotechnology (Santa Cruz, CA, USA). Antibodies for p-p70S6K (#GTX530304, Ser424), p-eIF-4E (#GTX50268, Ser209), and p-4E-BP1 (#GTX61987, Thr37) were purchased from Gene Tex Inc. (Irvine, CA, USA). Goat anti-rabbit (#NEFB812001EA) and anti-mouse (#NEF822001EA) secondary antibodies were purchased from PerkinElmer Biosciences (Boston, MA, USA). Mouse VEGF ELISA kit was purchased from Sigma Aldrich (St. Louis, MO, USA).

### 2.2. Cell Culture

The highly malignant, estrogen-receptor independent +SA mouse mammary epithelial cells were derived from an adenocarcinoma that developed spontaneously in a BALB/c female mouse [27, 28]. +SA cells were cultured as described previously [20, 29, 30]. Briefly, cells were maintained in serum-free defined Dulbecco's modified Eagle's medium (DMEM)/Ham's F12, supplemented with 5 mg/mL bovine serum albumin (BSA), 10 *μ*g/mL transferrin, 100 U/mL soybean trypsin inhibitor, 100 U/mL penicillin, 0.1 mg/mL streptomycin, and 10 *μ*g/mL insulin at 37°C, in an environment of 95% air and 5% CO_2_ in a humidified incubator. For subculturing, cells were washed twice with sterile Ca^+2^- and Mg^+2^-free phosphate buffered solution (PBS) and incubated with 0.05% trypsin containing 0.025% EDTA in PBS for 5 min at 37°C. Released cells were centrifuged and resuspended in serum containing media and counted using a hemocytometer.

### 2.3. Preparation of *δ*-Tocotrienol Oxazine Derivative, Compound **44**

**Figure 1:**
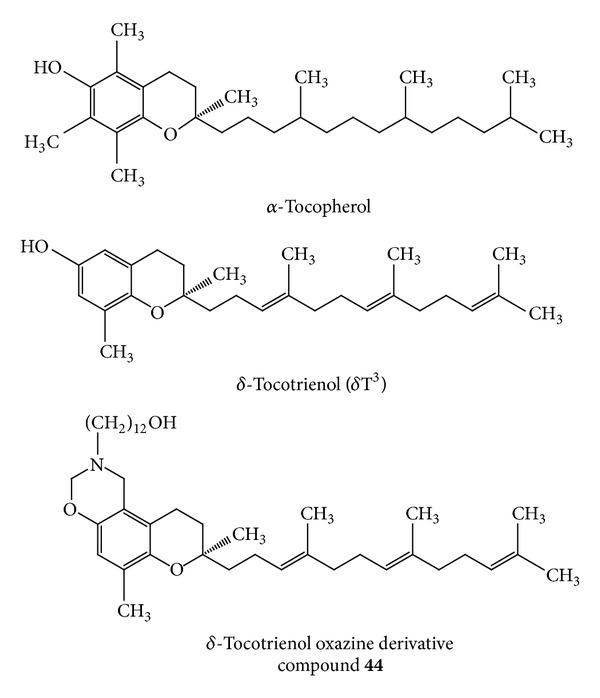
Chemical structures of *α*-tocopherol, *δ*-tocotrienol, and the *δ*-tocotrienol oxazine derivative, 12-((R)-6,8-dimethyl-8-((3E,7E)-4,8,12-trimethyltrideca-3,7,11-trienyl)-9,10-dihydrochromeno[5, 6-e] [1, 3]oxazin-2(1H, 3H, 8H)-yl)dodecan-1-ol)** (**compound** 44).**

Compound** 44** is the oxazine derivatives of *δ*-tocotrienol. Preparation, structural verification, and classification of tocotrienol oxazine derivatives, particularly compound** 44,** were previously described in detail [23, 24]. Based on results obtained from previous studies, compound** 44** was found to display the most potent anticancer activity in both cell culture and animal tumor models, as compared to its natural parent *δ*-tocotrienol compound [23, 24], and was therefore selected for further characterization of its effects on +SA mammary tumor cell compensatory response to CoCl_2_-induced hypoxia in both cell culture and syngeneic mouse mammary tumor models. Chemical structures of natural vitamin E isoforms, *α*-tocopherol and *δ*-tocotrienol, and the semisynthetic oxazine derivative of *δ*-tocotrienol are shown in Figure 1.

### 2.4. Experimental Treatments

The highly lipophilic natural and semisynthetic tocotrienols were first dissolved in DMSO and then suspended in a sterile 10% BSA stock solution as previously described [23, 24]. This stock solution was then used to prepare treatment media containing different concentrations of tocotrienols. Appropriate amounts of DMSO were then added to all media so that exposure to this agent was the same for all cells in a particular experiment. The final concentration of DMSO in any given experiment was always less than 0.1%. Dose- and time-response studies were conducted to examine treatment effects on CoCl_2_-induced hypoxic response in +SA mammary tumor cells grown in culture. +SA cells were suspended at a density of 5 × 10^3^ cells in 100 *µ*L control serum-free defined media and then seeded in each well of a 96-well culture plate and then returned to the incubator to allow cells to adhere to the bottom of the plate. On the following day, cells were divided into different treatment groups (6 wells/group), the original media were removed, and all wells received 100 *µ*L of their respective treatment media containing 0–300 *µ*M CoCl_2_, 0–2 *µ*M *δ*-tocotrienol, or 0–2 *µ*M *δ*-tocotrienol oxazine derivative (compound** 44**) alone or in combination. Since higher treatment doses of *δ*-tocotrienol or compound** 44** have previously been found to have antiproliferative and apoptotic effects on +SA tumor cells and significantly reduce viability [23, 24], a subeffective growth inhibitory dose (2 *µ*M) was chosen for use in these studies. Cells in all treatment groups were provided fresh media every other day throughout the experiment.

### 2.5. Measurement of Viable Cell Number

Viable cell number was determined using the 3-(4,5-dimethylthiazol-2yl)-2,5-diphenyl tetrazolium bromide colorimetric (MTT) colorimetric assay as described previously [20, 29, 30]. Briefly, at the end of a given culture period, media in all treatment groups were replaced with fresh control media containing 0.5 mg/mL MTT. After a 3 hr incubation period, the media were removed, MTT crystals were dissolved in DMSO (100 *μ*L/well), and optical density of each sample was measured at 570 nm on a microplate reader (SpectraCount, Packard BioScience Company, Meriden, CN). The numbers of cells/well was calculated against a standard curve prepared by plating various concentrations of cells, as determined by hemocytometer, at the beginning of each experiment.

### 2.6. Measurement of VEGF in Culture Media

For quantitative measurement of VEGF levels in culture media, enzyme-linked immunosorbent assay (ELISA) colorimetric analysis was used in this study following the manufacturer's instructions provided in the kit. Briefly, +SA mammary cells were seeded at a concentration of 2 × 10^4^ cells/well in 96-well culture plates and allowed to attach overnight. The following day, cells were divided into 4 treatment groups consisting of 3 replicates/group and consisted of the following: (a) vehicle-treated control, (b) 150 *µ*M CoCl_2_, (c) 2 *µ*M compound** 44**, (d) 150 *µ*M CoCl_2_ + 2 *µ*M compound** 44,** and they were exposed to their respective treatment for a 24 hr period. Afterwards, media from the different treatment groups were collected, and 100 *µ*L from each sample was added to the VEGF coated ELISA plate and incubated overnight at 4°C with gentle shaking. The next day, the assay solution was removed and all wells were rinsed 4 times with the wash solution provided in the kit, and then 100 *µ*L of biotinylated antibody solution was added to each well and incubated for 1 hr at room temperature with gentle shaking. Afterwards, the antibody solution was discarded and each well was again washed 4 times with wash solution, and then 100 *µ*L of streptavidin solution was added to each well and allowed to incubation at room temperature for 45 min with gentle shaking. Afterwards, the streptavidin solution was removed and all wells were again washed 4 times and then 100 *µ*L of 3,3′,5,5′-tetramethylbenzidine (TMB) substrate and incubated for 30 min in the dark at room temperature with gentle shaking. At the end of this time, 50 *µ*L of stop solution was added to each well. Color intensity in each well was measured at 450 nm using a microplate reader (SpectraCount, Packard BioScience Company). The concentration of VEGF in each sample was calculated against a standard curve provided in the kit.

### 2.7. Preparation of Nanoemulsions

Nanoemulsions were prepared using high-pressure homogenization techniques described previously [31–33]. Briefly, an individual vitamin E isoform or its derivative was mixed at a 1 : 1 (w/w) ratio with medium-chain triglycerides, and then the mixture was dissolved in chloroform to ensure homogeneity of the oil phase. Samples were then placed in a vacuum oven overnight to remove the chloroform by evaporation. In a separate vial, primary and secondary emulsifiers (0.12% Lipoid E80S and 0.05% Tween 80) were dispersed in deionized water to which 0.25% PEG_2000_-DSPE was added to form the aqueous phase of the nanoemulsion. Glycerol (2.25%) was then added to adjust tonicity. The two phases were then combined and passed through a high-pressure homogenizer (EmulsiFlex C3; Avestin Inc., Ottawa, Canada) for 25 cycles under a homogenization pressure of 170 MPa. The pH of the resulting nanoemulsions was then adjusted to 8 ± 0.05 using 0.1 N sodium hydroxide because previous studies have shown that lipid emulsions are most stable at pH values higher than 7.5 [31–33]. Previous studies have demonstrated that vitamin E compounds prepared for cell culture and nanoemulsion formulations remain very stable and retain their bioactivity for up to 6 months when stored at 4°C [32].

### 2.8. *In Vivo* Tumor Model and Study Design

Female BALB/c mice, 4–6 weeks of age, were purchased from Harlan Sprague-Dawley (Indianapolis, IN, USA) and housed in plastic cages in a temperature-regulated (24 ± 0.5°C) and light-controlled (12 h light/12 h dark) room and allowed standard laboratory mouse chow and water* ad libitum.* All experiments were approved by the Institutional Animal Care and Use Committee (Animal Welfare Assurance Number A3641-01). At the time of tumor cell inoculation, animals were anesthetized with an i.p.injection of ketamine/xylazine (10 mg ketamine: 1 mg xylazine/mL saline; Henry Schein, Inc, Melville, NY) at a dose of 0.1 mL/10 g body weight. A small incision was made in the skin along the midline of the abdomen, and 1 × 10^6^ +SA mammary cells suspended in 100 *µ*L 0.05 M PBS was injected into the abdominal fat pad of the number 4 left mammary gland and the incision was then closed. Animals were allowed to recover and then returned to their cage. Mice developed palpable mammary tumors within 4–6 weeks after implantation. When tumors reached 4-5 mm in diameter, mice were randomly divided into 4 experimental treatment groups (8 mice/group) that included (a) untreated control; (b) *α*-tocopherol; (c) *δ*-tocotrienol; (e) *δ*-tocotrienol oxazine derivative (compound** 44)**. Nanoemulsion treatments were administered by intralesional injection at a concentration of 120 *μ*g/20 *μ*L for *α*-tocopherol, *δ*-tocotrienol, and compound** 44**; the control group received similar treatment with a vehicle-filled nanoemulsion preparation. Mice received treatment injections every other day for 11 days (total of six intralesional injections). Tumor size was determined daily for each tumor by measuring the two largest perpendicular diameters as measured by vernier calipers as described previously [23, 34] and tumor volume was calculated using the following formula:

(1) $$\begin{array}{c} \text{Volume}\left({\text{cm}}^{3}\right)=\frac{\text{length}\left(\text{cm}\right)\times \;{\text{width}}^{2}\left(\text{cm}\right)}{2}. \end{array}$$

At the end of the 11-day treatment period, mice were sacrificed and mammary tumors were removed under aseptic conditions, weighed, and then immediately frozen at −80°C for subsequent Western blot analysis.

### 2.9. Western Blot Analysis

A small portion (5–10 mg) of cell/tumor lysate from all treatment groups was homogenized in Laemmli buffer that consisted of 0.5 M Tris base, 10% sodium dodecyl sulfate (SDS), 2-*β*-mercaptoethanol, and glycerol containing 100 *μ*M sodium orthovanadate, a protease inhibitor [35]. For tumor samples, lysates were incubated at 4°C for 30 min and mixed intermittently using a vortex and then centrifuged at 12,000 ×g for 15 min at 4°C. Supernatant was collected and protein concentration in each sample was determined using Bio-Rad protein assay kit (Bio Rad, Hercules, CA, USA). Equal amounts of protein (30–40 *μ*g) of each sample were then subjected to electrophoresis through 10–15% SDS polyacrylamide mini-gels. Each gel was then equilibrated in transfer buffer and trans-blotted at 30 V for 12–16 h at 4°C to a polyvinylidene fluoride membrane (PerkinElmer Lifesciences, Wellesley, MA, USA) in a Trans-Blot Cell (Bio-Rad Laboratories) according to the methods of Towbin et al. [36]. Nonspecific antibody binding sites were blocked by incubating trans-blotted membranes in 2% BSA in 10 mM Tris-HCl containing 50 mM NaCl and 0.1% Tween 20, pH 7.4 (TBST) for 2 h. Afterwards, membranes were washed 5 times with TBST followed by incubation with specific primary antibodies raised against PI3K, p-Akt, mTOR, p-mTOR, HIF-1*α*, p-p70S6K, p-eIF-4E, p-4E-BP1, MEK, p-MEK 1/2, ERK1, ERK2, p-ERK 1/2, and *α*-tubulin diluted to 1 : 3000 in 2% BSA in TBST for overnight at 4°C. Membranes were then washed 5 times in TBST and incubated with respective horseradish peroxide-conjugated secondary antibody diluted 1 : 3000 to 1 : 5000 in 2% BSA in TBST for 1 hr at room temperature and then washed 3 times with TBST. Specific target protein bands on each membrane were then visualized by chemiluminescence according to the manufacturer's instructions (Pierce, Rockford, IL, USA). Images of protein bands from all treatment groups were acquired using the Syngene Imaging System (Beacon House, Nuffield Road, Cambridge, UK). The visualization of *α*-tubulin was used to ensure equal sample loading in each lane. Scanning densitometric analysis was performed with Kodak molecular imaging software version 4.5 (Carestream Health Inc, Rochester, NY, USA). All experiments were repeated at least 3 times and a representative Western blot image from each experiment is shown in the figures.

### 2.10. Statistics

Statistical differences between various treatment groups in cell viability, tumor growth, ELISA, and Western blot scanning densitometric analyses were determined using analysis of variance, followed by Duncan's multiple range test. A difference of *P* < 0.05 was considered statistically significant as compared with the vehicle-treated control group or as defined in the figure legends.

## 3. Results

### 3.1. Cytotoxic Effect of CoCl_2_ on +SA Mammary Tumor Cells

**Figure 2:**
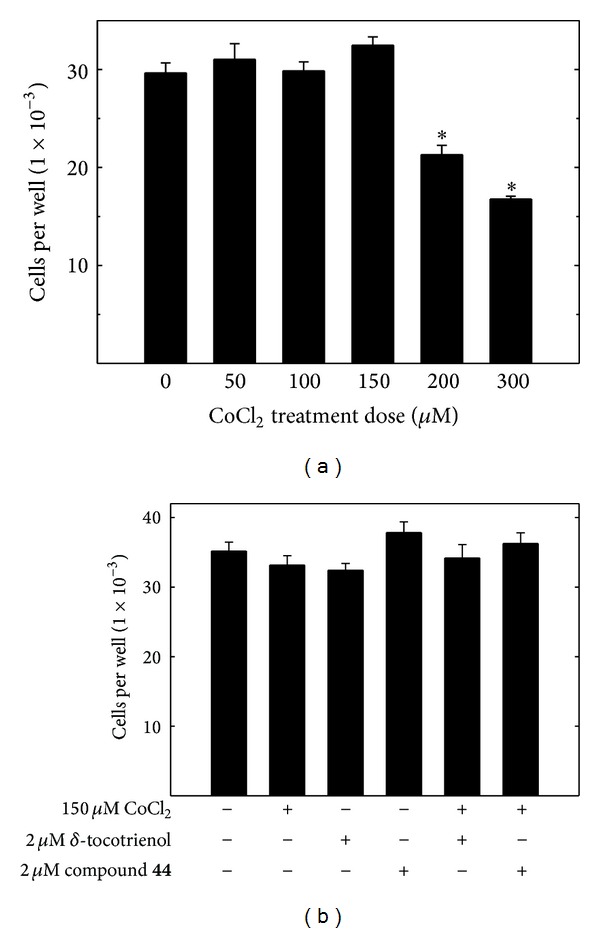
(a) Dose-response effects of CoCl_2_ on mouse +SA mammary tumor cell viability. +SA cells were initially plated at a density of 5 × 10^3^ cells/well in 96-well culture plates (8 replicates/group) and exposed to 0–300 *µ*M CoCl_2_ for a 24 hr incubation period. Afterwards, viable cell number was determined using the MTT assay. (b) Effects of 150 *µ*M CoCl_2_ (noncytotoxic dose) alone and in combination with subeffective antiproliferative doses (2 *µ*M) of *δ*-tocotrienol or the *δ*-tocotrienol oxazine derivative, compound** 44**, on +SA mammary tumor cell viability. +SA cells were initially plated at a density of 5 × 10^3^ cells/well in 96-well culture plates (8 replicates/group) and their respective treatments for a 24 hr incubation period. Afterwards, viable cell number was determined using the MTT assay. Vertical bars indicate mean viable cell number ± SEM. *P < 0.05 compared to the vehicle-treated control group.

A 24 hr treatment exposure to 0–150 *µ*M CoCl_2_ was found to have little or no effect on +SA cell viability as compared to the vehicle-treated control group (Figure 2(a)). However, similar treatment with 200–300 *µ*M CoCl_2_ significantly decreased +SA cell viability (Figure 2(a)). Additional studies showed that a 24 hr treatment exposure to 2 *µ*M *δ*-tocotrienol or 2 *µ*M of the *δ*-tocotrienol oxazine derivative, compound** 44**, alone or in combination with 150 *µ*M CoCl_2 _was also not found to have any significant effect on +SA mammary tumor cells viability as compared to the vehicle-treated control group (Figure 2(b)).

### 3.2. Effects of CoCl_2_-Induced Hypoxic Response on HIF-1*α* Expression

**Figure 3:**
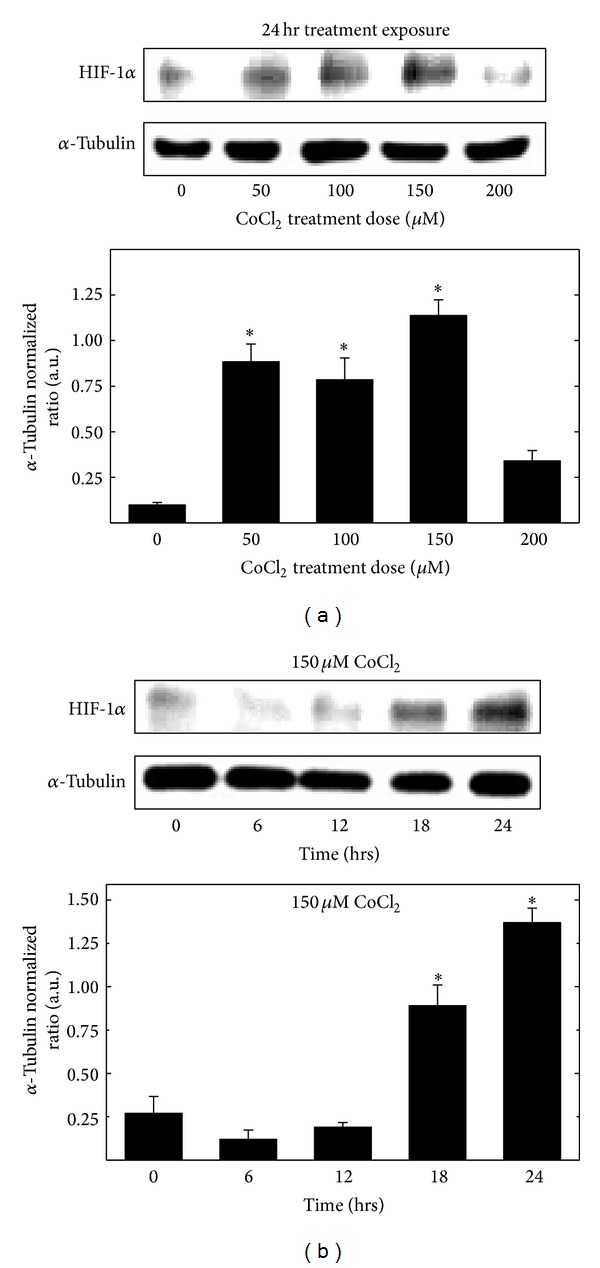
(a) Dose-response and (b) time-response effect of CoCl_2_ on HIF-1*α* levels in +SA mammary cancer cells grown in culture. +SA cells were seeded at concentration of 1.5 × 10^6^ in 100 mm culture dishes and allowed to attach overnight. The following day, cells were divided into treatment groups and exposed to various concentrations of CoCl_2_ for 0–24 hr incubation period. Afterwards, cells were isolated with trypsin, and whole cell lysates were prepared for Western blot analysis. Scanning densitometric analysis was performed on all blots done in triplicate and the integrated optical density of each band was normalized with corresponding *α*-tubulin, as shown in the bar graphs below their respective Western blot image. Vertical bars indicate the normalized integrated optical density of bands visualized in each lane ± SEM. *P < 0.05 as compared to the vehicle-treated controls.

Dose- and time-dependent studies were conducted to determine the effects of CoCl_2_ treatment on HIF-1*α* levels in +SA mammary tumor cells. Treatment with 0–150 *µ*M CoCl_2_ resulted in a dose-responsive increase in HIF-1*α* levels after a 24 hr incubation period, whereas treatment with 200 *µ*M CoCl_2_ attenuated this response (Figure 3(a)). Based on these findings and the results in Figure 2(a) that showed treatment with 200–300 *µ*M CoCl_2_ decreased +SA cell viability, a treatment dose of 150 *µ*M CoCl_2_ was chosen for subsequent experimentation because this dose induced a robust hypoxic response, as indicated by a large increase in HIF*α*-1 levels, without causing cytotoxic effects on +SA tumor cells growth or viability (Figure 3(a)). Treatment with 150 *µ*M CoCl_2_ displayed a time-responsive increase in HIF-1*α* levels that peaked at 24 hr after the initiation of treatment (Figure 3(b)).

### 3.3. *δ*-Tocotrienol and Its Oxazine Derivative Effects on CoCl_2_-Induced HIF-1*α* Expression

**Figure 4:**
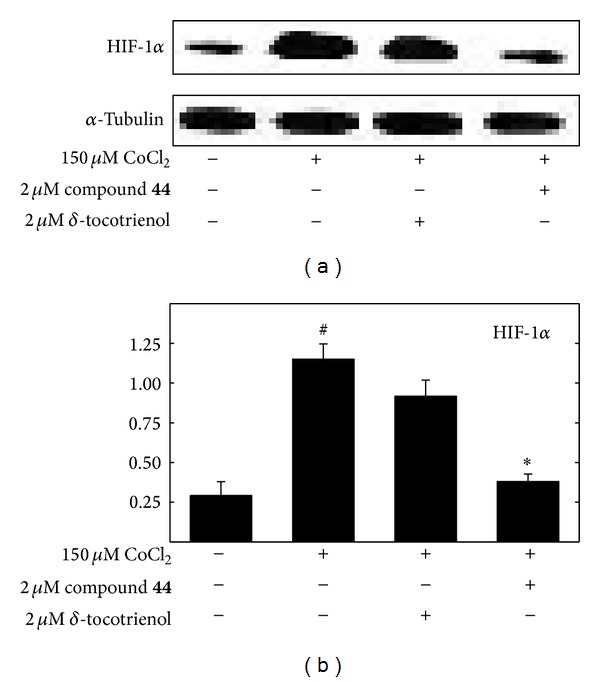
(a) Effects of 150 *µ*M CoCl_2_ (hypoxic, but not cytotoxic dose) alone and in combination with subeffective doses (2 *µ*M) of *δ*-tocotrienol or the *δ*-tocotrienol oxazine derivative, compound** 44**, on HIF-1*α* levels in +SA mammary tumor cells. +SA cells were seeded at concentration of 1.5 × 10^6^ in 100 mm culture dishes and allowed to attach overnight. The following day, cells were divided into groups and exposed to their respective treatments for a 24 hr incubation period. Afterwards, whole cell lysates were prepared for Western blot analysis. (b) Scanning densitometric analysis was performed on all blots done in triplicate and the integrated optical density of each band was normalized with corresponding *α*-tubulin, as shown in the bar graphs below their respective Western blot image. Vertical bars indicate the normalized integrated optical density of bands visualized in each lane ± SEM. ^#^ *P* < 0.05 compared to the vehicle-treated control group. *P < 0.05 as compared to the hypoxic group treated with CoCl_2_ alone.

Exposure for 24 hr to 150 M CoCl_2 _significantly increased the HIF-1 expression in +SA mammary tumor cells (Figures 4(a) and 4(b)). However, combined treatment with 2 *µ*M *δ*-tocotrienol was found to attenuate, while combined treatment with the *δ*-tocotrienol oxazine derivative, compound** 44**, significantly inhibited CoCl_2_-induced HIF-1 protein expression (Figures 4(a) and 4(b)).

### 3.4. *δ*-Tocotrienol Oxazine Derivative Blockade of CoCl_2_-Induced Hypoxic Response Effects on PI3K/Akt/mTOR Signaling

**Figure 5:**
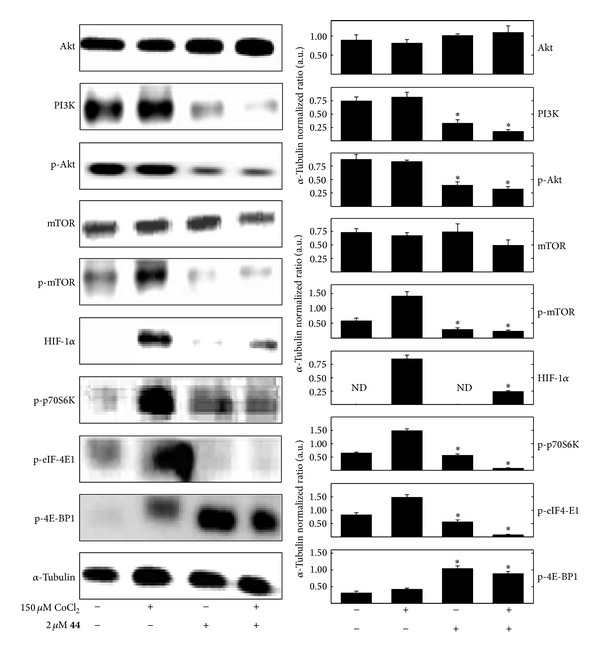
Effects of 150 *µ*M CoCl_2 _(hypoxic, but not cytotoxic dose) alone and in combination with compound** 44 **(**44**), on mitogenic/survival signaling and hypoxic response marker protein levels in +SA mammary tumor cells. +SA cells were seeded at concentration of 1.5 × 10^6^ in 100 mm culture dishes and allowed to attach overnight. The following day, cells were divided into groups and exposed to their respective treatments for a 24 hr incubation period. Afterwards, whole cell lysates were prepared for Western blot analysis for Akt, PI3K, phospho-Akt (p-Akt, Ser473), mTOR, phospho-mTOR (p-mTOR, Ser2448), HIF-1*α*, phospho-p70S6K (p-p70S6K, Ser424), phospho-eIF-4E1 (p-eIF-4E1, Ser209), and phospho-4E-BP1 (p-4E-BP1, Thr37). Scanning densitometric analysis was performed on all blots done in triplicate and the integrated optical density of each band was normalized with corresponding *α*-tubulin, as shown in the bar graphs below their respective Western blot image. Vertical bars indicate the normalized integrated optical density of bands visualized in each lane ± SEM. *P < 0.05 as compared to the hypoxic CoCl_2_-treated controls.

Treatment for 24 hr to 150 *µ*M CoCl_2_ alone had little or no effect on total levels of Akt, PI3K, and mTOR or phospho-Akt but did cause a relatively large increase in phospho-mTOR, HIF-1*α*, phospho-p70S6K, phospho-elF-4E1 levels in +SA mammary tumor cells, as compared to the vehicle-treated control group (Figure 5). Treatment with 2 *μ*M compound** 44** alone had little or no effect on total Akt, mTOR, HIF-1*α*, phospho-p70S6K, or phospho-eIF-4E1 levels but caused a relatively large decrease in total PI3K, phospho-Akt, and phospho-mTOR and corresponding increase in phospho-4E-BP1 levels in +SA cells as compared to the vehicle-treated control group (Figure 5). Combined treatment with these same doses of CoCl_2_ and compound** 44 **attenuated the hypoxic response characterized by a blockade of CoCl_2_-induced increases in phospho-mTOR, HIF-1*α*, phospho-p70S6K, and phospho-eIF-4E1 (Figure 5). In addition, phospho-4E-BP1 levels remained significantly increased in the combined treatment group as compared to the group treated with CoCl_2_ alone (Figure 5).

### 3.5. *δ*-Tocotrienol Oxazine Derivative Blockade of CoCl_2_-Induced Hypoxic Response Effects on MAPK Signaling

**Figure 6:**
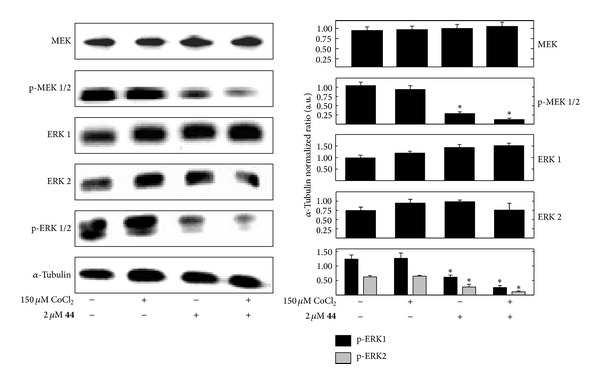
Effects of 150 *µ*M CoCl_2_ (hypoxic, but not cytotoxic dose) alone and in combination with compound** 44 **(**44**), on MAPK cascade signaling proteins in +SA mammary tumor cells. +SA cells were seeded at concentration of 1.5 × 10^6^ in 100 mm culture dishes and allowed to attach overnight. The following day, cells were divided into groups and exposed to their respective treatments for a 24 hr incubation period. Afterwards, whole cell lysates were prepared for Western blot analysis or MEK, phospho-MEK 1/2 (p-MEK 1/2, Ser221/217), ERK1, ERK2, and phospho-ERK 1/2 (p-ERK 1/2, Thr202/Tyr204). Scanning densitometric analysis was performed on all blots done in triplicate and the integrated optical density of each band was normalized with corresponding *α*-tubulin, as shown in the bar graphs below their respective Western blot image. Vertical bars indicate the normalized integrated optical density of bands visualized in each lane ± SEM. *P < 0.05 as compared to the hypoxic CoCl_2_-treated control group.

Following a 24 hr treatment exposure to 150 *µ*M CoCl_2_ alone +SA mammary tumor cells displayed little change in their the relative levels of MEK, phospho-MEK1/2, ERK1, ERK2, or phospho-ERK1/2 as compared to the +SA mammary tumor cells in the vehicle-treated control group (Figure 6). Treatment with 2 *μ*M compound** 44** alone had little or no effect on total MEK, ERK1, and ERK2 levels but did cause a relatively large decrease in phospho-MEK1/2 and phospho-ERK1/2 levels, as compared to +SA cells in the vehicle-treated control group (Figure 6). Combined treatment with these same doses of CoCl_2_ and compound** 44 **produced similar effects as those observed in cells treated with compound** 44** alone (Figure 6).

### 3.6. *δ*-Tocotrienol Oxazine Derivative Blockade of CoCl_2_-Induced Hypoxic Response Effects on VEGF Production

**Figure 7:**
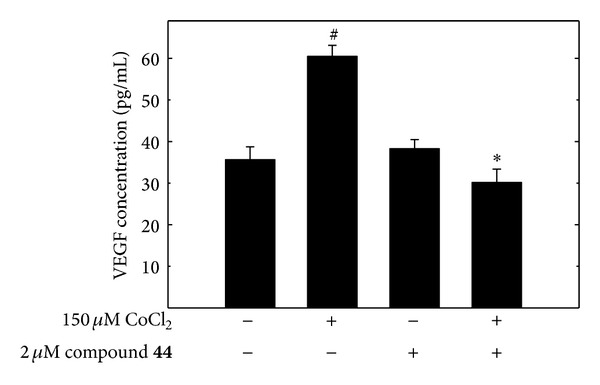
ELISA quantification of VEGF protein levels in the culture media following +SA mammary tumor cells exposed to 150 *µ*M CoCl_2_ alone and in combination with compound** 44**. +SA cells were plated at a density of 5 × 10^3^ cells/well in 96-well culture plates (6 replicates/group) in 96-well tissue culture plates and allowed to attach overnight. The next day, cells were divided into different groups and exposed to their respective treatments for a 24 hr incubation period. Afterward, cell media from wells in each treatment group and added to VEGF antibody coated 96-well plates for ELISA analysis. Vertical bars indicate mean VEGF levels (pg/mL) ± SEM. ^#^ *P* < 0.05 compared to the vehicle-treated control group. *P < 0.05 as compared to the hypoxic group treated with CoCl_2 _alone.

After a 24 hr treatment exposure to 150 *µ*M CoCl_2_, +SA mammary tumor cell synthesis of VEGF was significantly increased as compared to cells in the vehicle-treated control group (Figure 7). Treatment with 2 *μ*M of the *δ*-tocotrienol oxazine derivative, compound** 44**, alone had little or no effect on +SA mammary tumor cell VEGF synthesis as compared to the vehicle-treated control group (Figure 7). However, combined treatment of compound** 44 **resulted in a blockade of the CoCl_2_-dependent increase in VEGF synthesis in +SA cells (Figure 7).

### 3.7. *In Vivo* Anticancer Effects of *δ*-Tocotrienol and Its Oxazine Derivative and of Tumor HIF-1*α* Levels and Akt/mTOR Signaling

**Figure 8:**
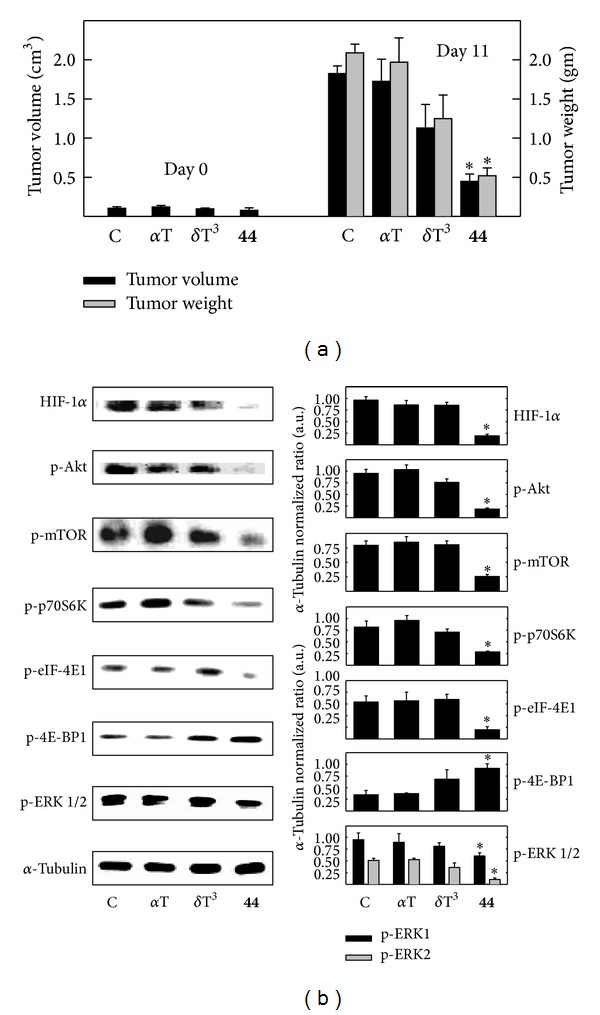
(a) Average +SA tumor volume in the treatment groups at the start (Day 0) and end (Day 11) of the treatment period. +SA mammary cells (1 × 10^6^) suspended in 100 *μ*L of 0.05 M PBS were injected into the number 4 abdominal mammary fat pad of syngeneic female BALB/c mice. Once tumors reached 4-5 mm in diameter, mice were divided into different treatment groups (8 mice/group) and treated with intralesional injections of lipid nanoemulsion formulations of *α*-tocopherol (*α*T), *δ*-tocotrienol (*δ*T^3^), or *δ*-tocotrienol oxazine derivative, compound** 44** (**44**), at a dose of 0–120 *μ*g/20 *μ*L every other day throughout the experimental period. The untreated control group (C) was added to ensure that intralesional injection of the *α*-tocopherol nanoemulsion did not influence tumor growth in a nonspecific manner. Data points indicate the average tumor volume (cm^3^± SEM) for 8 mice/group ± SEM in each treatment group. *P < 0.05, as compared with the *α*-tocopherol-treated negative control group. (b) Western blot analysis of HIF-1*α*, phospho-Akt (p-Akt, Ser473), phospho-mTOR (p-mTOR, Ser2448), phospho-p70S6K (p-p70S6K, Ser424), phospho-eIF-4E1 (p-eIF-4E1, Ser209), phosphor-4E-BP1 (p-4E-BP1, Thr37), and phospho-ERK1/2 (p-ERK1/2, Thr202/Tyr204) in +SA mammary tumors grown in syngeneic BALB/c mice exposed to the various treatments. Lysates prepared from each tumor were separated by polyacrylamide gel electrophoresis (40 *μ*g/lane) followed by Western blot analysis. *α*-Tubulin was visualized to ensure equal sample loading in each lane. Each Western blot is a representative image of data obtained for experiments that were repeated at least three times. Scanning densitometric analysis was performed on all blots in triplicate and the optical density of each band was normalized with that of the corresponding *α*-tubulin, as shown in bar graphs. Vertical bars indicate the normalized integrated optical density of bands visualized in each lane ± SEM. *P < 0.05, as compared with the *α*-tocopherol-treated negative control group. C: Untreated control; *α*-T: *α*-tocopherol-treated negative control; *δ*T^3^: *δ*-tocotrienol;** 44**: *δ*-tocotrienol oxazine derivative, compound** 44**.

The effects *δ*-tocotrienol and its oxazine derivative, compound** 44,** on +SA mammary tumor cell compensatory response to CoCl_2_-induced hypoxia were further characterized in an* in vivo* animal tumor model. Female BALB/c mice bearing syngeneic +SA mammary tumors were divided into different treatment groups and treated with nanoemulsion preparations that contained equal concentrations of *α*-tocopherol (negative control), *δ*-tocotrienol, compound** 44,** and an untreated control group added to ensure that intralesional injection of the *α*-tocopherol nanoemulsion did not influence +SA mammary tumor growth through a nonspecific mechanism. +SA mammary tumor growth in mice treated with the nanoemulsion preparation containing *α*-tocopherol displayed continuous and rapid growth throughout the 11-day treatment period, and this growth was not found to differ significantly from tumors grown in the untreated control group (Figure 8(a)). +SA mammary tumor growth in mice treated with *δ*-tocotrienol nanoemulsion preparation was reduced as compared to the *α*-tocopherol treated negative control group, but this inhibition was not found to be statistically significant (Figure 8(a)). However, +SA mammary tumor growth in mice treated with the *δ*-tocotrienol oxazine derivative was significantly reduced as compared to tumor growth in the *α*-tocopherol-treated negative control group and as compared to its natural *δ*-tocotrienol parent compound (Figure 8(a)). Animal body weight was not found to be significantly different between any of the different treatment groups at any time during the 11-day treatment period (data not shown).

Western blot analysis showed that +SA mammary tumors grown in the untreated control group or the *α*-tocopherol negative treatment control group displayed a relatively high expression of HIF-1*α*, phospho-Akt, phospho-ERK1/2, phospho-mTOR, phospho-p70S6K, and phospho-eIF-4E1 levels and relatively low levels of phospho-4E-BP1 (Figure 8(b)). Treatment with nanoemulsions containing natural *δ*-tocotrienol had only a slight effect on the expression of these signaling proteins (Figure 8(b)). In contrast, +SA mammary tumors from mice treated with the *δ*-tocotrienol oxazine derivative, compound** 44**, displayed a relatively large and significant decrease in HIF-1*α*, phospho-Akt, phospho-ERK1/2, phospho-mTOR, phospho-p70S6K, and phospho-eIF-4E1 levels and a corresponding increase in the levels of phospho-4E-BP1 (Figure 8(b)).

## 4. Discussion

Experimental findings in the present study demonstrate that a hypoxic response can be induced in +SA mammary tumor cells with treatment of CoCl_2_. Results showed that a 24 hr exposure to 150 *µ*M CoCl_2_ causes a large increase in HIF-1*α* levels, and combined treatment with compound** 44 **attenuated this response. The CoCl_2_-induced hypoxic response in +SA cells was also associated with a large increase in Akt/mTOR signaling, activation of their downstream targets p70S6K, and eIF-4E1, and a significant increase in VEGF production. However, this response was also blocked by combined treatment with 2 *µ*M compound** 44**. Furthermore, these findings were confirmed with* in vivo* studies in BALB/c mice implanted with syngeneic +SA mammary tumors. Taken together, these findings demonstrate that treatment with the *δ*-tocotrienol oxazine derivative, compound** 44**, significantly attenuates +SA mammary tumor cell compensatory response to hypoxia and suggests that this compound may provide benefit in the treatment of rapidly growing solid breast tumors.

Tumor growth can be broadly divided into two stages. The first stage consists of rapid growth that increases tumor bulk. The second stage is characterized by slow growth due to insufficient vascularization that is unable to adequately provide oxygen and nutrients to the tumor. Large tumor size can cause a reduction in the amount of oxygen that diffuses into the inner tumor mass, thereby producing hypoxic conditions [37]. Hypoxia leads to certain physiological changes in tumor cell microenvironment as a response to compensate for the lack of optimum oxygen and nutrients to the inner regions of tumor mass. Furthermore, hypoxic conditions have been associated with the promotion of tumor heterogeneity and progression, resulting in a poor patient prognosis [38]. Increased expression of the transcription factor, HIF-1*α*, in tumor cells is a hallmark of hypoxia, and HIF-1*α* plays a central role promoting tumor cell survival and growth during hypoxic conditions by changing the expression profile of many genes particularly angiogenic factors such as VEGF [39, 40]. HIF-1*α* stimulates the activation of various signaling pathways involved in metabolic adaptation, angiogenesis, cell growth, differentiation, survival, and apoptosis [41].

Results in the present study showed that treatment with the semisynthetic *δ*-tocotrienol oxazine derivative, compound** 44**, was effective in blocking +SA mammary tumor cell compensatory response to hypoxia, by suppressing HIF-1*α* expression, as well as the activation of Akt/mTOR and MAPK pathways, and VEGF production. In contrast, treatment with the natural parent compound, *δ*-tocotrienol, was found to be less potent in blocking +SA mammary tumor hypoxic response. Studies have shown that hypoxic conditions modulate HIF-1*α* levels by decreasing its degradation rather than synthesis [6]. Moreover, activation of Akt/mTOR pathway appears to stimulate accumulation and decrease degradation of HIF-1*α* in hypoxic tumor cells [42]. One of the downstream targets of Akt is mTOR, which is a serine/threonine kinase that is part of the mTORC1 complex [15]. By integrating both intracellular and extracellular signals, mTOR serves as a key factor for cell metabolism, growth, proliferation, and survival during hypoxic conditions [15]. It is also interesting to note that all cell culture experiments conducted in the present study administered CoCl_2_ and experimental treatments at the same time as cotreatments, and it remains to be determined if the same results would be obtained if cells were pretreated with CoCl_2_ prior to treatment exposure. Although the results obtained from animal tumor studies would suggest that similar treatment effects would be observed, Although the results obtained from animal tumor studies would suggest that similar treatment effects would still be observed if hypoxia was induced prior to drug exposure, since preexisting hypoxic conditions were found in the tumors, further studies are still required to resolve this question.

Previous studies have shown that although low oxygen level is the primary influence involved in regulating HIF-1*α* expression, other signaling pathways like PI3K/Akt increase the amplitude of HIF-1*α* expression by increased translation of HIF-1*α* mRNA into protein [43, 44]. Additional studies suggest that mTOR is the upstream regulator of HIF-1*α* in cancer cells [45]. Along with Akt, mTOR also serves as an amplifier, rather than a trigger, for HIF-1*α* activation [46]. Results from the present study show that the CoCl_2_-induced hypoxic response in +SA mammary tumor cells caused a large increase in Akt/mTOR signaling proteins associated with promoting HIF-1*α* expression, and compound** 44 **blocked this response. These findings indicate that this *δ*-tocotrienol oxazine derivative attenuates the activation of signaling pathways in +SA cells associated with mediating tumor cell compensatory response to hypoxia.

Activation of mTOR was also found to lead to the activation/deactivation of downstream targets, including p70S6kinase, eIF-4E1, and 4E-BP1 proteins. When 4E-BP1 is highly phosphorylated, it easily binds to eIF-4E1 to inhibit its activity [16]. Treatment with compound** 44** was found to inhibit the activation of mTOR downstream target proteins p70S6K and eIF-4E1 and inhibit HIF-1*α* expression. Specifically, treatment with compound** 44** significantly increased phosphorylation of 4E-BP1 and this effect was associated with a corresponding reduction in phosphorylated p70S6K and eIF-4E1 levels in these tumor cells.

Evidence has been provided in other studies indicating that activation of ERK1/2 (p42/p44) in the MAPK pathway also plays an important role in phosphorylation and activation of HIF-1*α* [19]. Studies have shown that p42/p44 ERK activity is responsible for increasing transcriptional activity of HIF-1*α*, resulting from the cross-talk integration between hypoxic and growth factor signals [19]. Results in the present study show that treatment with compound** 44** significantly decreased the phosphorylation and activation of ERK1/2 and this effect may also play a role in causing a corresponding decrease in HIF-1*α* and VEGF levels. VEGF synthesis is strongly stimulated by HIF-1*α* mediated gene transcription [12]. VEGF plays an important role in promoting angiogenesis in tumor tissues and is responsible for tumor cell growth under hypoxic conditions [12, 47]. Results showed that VEGF synthesis was greatly enhanced in hypoxic +SA mammary tumor cells, and combined treatment with compound** 44** blocked this compensatory response to hypoxia. These findings strongly suggest that this *δ*-tocotrienol oxazine derivative may provide significant benefit in the treatment of rapidly growing solid breast tumors.

## 5. Conclusion

Results in these studies demonstrate that exposure to 150uM CoCl2 cause a hypoxic response in +SA mammary tumor cells grown in culture, as evidenced by the large increased expression of the hypoxia-inducible factor, HIF-1*α*, without causing significant adverse effects on +SA cell viability or growth. Furthermore, CoCl_2_-induced increased expression of HIF-1*α* in +SA cells was attenuated by combined treatment with *δ*-tocotrienol or the *δ*-tocotrienol oxazine derivative, compound** 44**. Additional studies showed that CoCl_2_-induced hypoxia in +SA tumor cells was also associated with a relatively large increase in Akt/mTOR activation and their downstream targets p70S6kinase and eIF-4E1 and a significant increase in VEGF production, and combined treatment with compound** 44** blocked this response. Additional studies showed that +SA mammary tumors grown in syngeneic mice also displayed elevated HIF-1a levels and enhanced Akt/mTOR, p70S6K, and eIF-4E1 activation, and treatment with compound 44, but not its parent compound, *δ*-tocotrienol, blocked this compensatory response to hypoxic conditions. +SA mammary tumor cells respond by activating mitogenic and survival signaling pathways and increase production of VEGF in order to compensate for a reduction in oxygen availability, and this compensatory response to hypoxia can be significantly attenuated by treatment with compound** 44**. Taken together these findings suggest that treatment with the *δ*-tocotrienol oxazine derivative, compound** 44**, may provide some benefit in the treatment of rapidly growing tumors by blocking compensatory mechanisms that promote tumor survival and growth during hypoxic conditions. Additional studies are still required to further characterize these antagonistic effects of compound** 44** on tumor cell compensatory response to hypoxia in other breast cancer cell types, as well as other types of cancer cells.
